# Analytical Validation of MyProstateScore 2.0

**DOI:** 10.3390/diagnostics15070923

**Published:** 2025-04-03

**Authors:** Jacob I. Meyers, Tabea M. Schatz, Cameron J. Seitz, Rachel Botbyl, Bradley S. Moore, Bill G. Crafts, John R. Kitchen, Spencer Heaton

**Affiliations:** Lynx Dx, Ann Arbor, MI 48108, USA; tabea@lynxdx.com (T.M.S.); cameron@lynxdx.com (C.J.S.); rachel.botbyl@emory.edu (R.B.); bradleymoore@lynxdx.com (B.S.M.); bill@lynxdx.com (B.G.C.); john@lynxdx.com (J.R.K.); spencer@lynxdx.com (S.H.)

**Keywords:** prostate cancer, MyProstateScore 2.0, urinary biomarkers, analytical validation

## Abstract

**Background/Objectives**: Prostate cancer (PCa) is a leading cause of cancer-related deaths among men, with early detection playing a crucial role in improving outcomes. MyProstateScore 2.0 (MPS2), a novel urinary biomarker test, predicts clinically significant PCa to reduce invasive biopsy procedures. This study evaluates the analytical performance of MPS2 using both a post-digital rectal exam (DRE) and non-DRE urine samples. **Methods**: We assessed the reproducibility, precision, and detection limits of the eighteen MPS2 analytes. Analytical parameters including the linear range, upper and lower limits of quantification (ULOQ and LLOQ), and interference from substances commonly present in urine were evaluated. The reproducibility of the MPS2 scores was evaluated across post-DRE and non-DRE clinical urine samples. **Results**: MPS2 analytes demonstrated high linearity (R^2^ ≥ 0.975) across defined quantification ranges, with PCR efficiencies of 97–105%. The limits of detection (LOD) ranged from 40 to 160 copies/reaction, while the ULOQ was determined to be 10^6^–10^7^ copies/reaction for each analyte. Precision studies showed intra-run, inter-run, and inter-instrument standard deviations ≤0.5 Crt. Among the 12 potential interfering substances, only whole blood affected the performance of MPS2. The reproducibility of the MPS2 scores was consistent across post-DRE and non-DRE urine samples, meeting the acceptance criteria. **Conclusions**: The analytical validation confirms that MPS2 is robust and reliable in detecting biomarkers for clinically significant PCa. These findings, coupled with previous clinical validations, support the clinical use of MPS2 as a non-invasive diagnostic tool.

## 1. Introduction

Prostate cancer (PCa) is the most common cancer and the second leading cause of cancer deaths for men in the United States [1]. Survival is strongly associated with the stage at diagnosis, with a 5-year relative survival rate of 96.1% for localized disease versus 32.3% for distant metastasized disease [2]. This highlights the importance for early detection; however, routine screening for PCa remains controversial. Prostate specific antigen (PSA) is the most commonly used biomarker for PCa screening; however, routine PSA screening has a USPSTF rating of C for men 50–69 and D for men 70+, indicating an unclear benefit [3]. In fact, PSA screening has shown little to no improvement in PCa mortality and is associated with increased harm resulting from unnecessary prostate biopsies [4,5,6]. As such, the need remains for a PCa screening test with the ability to improve the early detection of clinically significant PCa while minimizing the risk of unnecessary biopsy and associated complications.

Urinary biomarkers have emerged as a promising method for the improved detection of clinically significant PCa (csPCa) [7]. Among these is MyProstateScore 2.0 (MPS2), a novel PCa risk prediction test which utilizes 18 prostate cancer-specific biomarkers to improve the predictive accuracy for GG ≥ 2 PCa. Using urine collected following a digital rectal exam (DRE), the test had a strong biopsy rule-out performance in a clinical validation study with a sensitivity of 89–94% and negative predictive values of 95–99% for csPCa, thus providing superior diagnostic accuracy to previously available urinary biomarker tests [8]. In comparison to PSA and PCPT, MPS2 identified up to 405 unnecessary biopsies per 1000 patients vs. 108 and 198 unnecessary biopsies for PSA and PCPT [8]. More recently, MPS2 was clinically validated using non-DRE urine, providing options for at-home sample collection. The test had a similarly strong biopsy rule-out performance with a sensitivity of 91–94% and negative predictive values of 92–96% [9]. Additionally, MPS2 was shown to have 95% sensitivity and 98% NPV to rule-out more advanced-grade group 3 prostate cancer [9]. These dual validations provide versatile options for urologists, who can choose whether or not to perform a DRE prior to sample collection based on their preferred clinical workflow.

The aim of this study is to validate the analytical performance of MPS2 on post-DRE and non-DRE urine. This was evaluated by measuring the reproducibility of MPS2 analyte detection and MPS2 scores across relevant technical parameters and in the presence of potential interfering substances.

## 2. Materials and Methods

### 2.1. RNA Extraction and Reverse Transcription

Optimized RNA extraction and reverse transcription protocols were individually developed for urine collected following DRE or without prior DRE using commercially available kits. Briefly, RNA was extracted from 0.5 mL of post-DRE urine (ThermoFisher Scientific, Waltham, MA, USA, Cat. No. A27828) on a KingFisher^TM^ Flex Purification System. Extracted RNA was reverse transcribed into cDNA (ThermoFisher Scientific, Cat. No. 11756050). Similarly, RNA was extracted from 5 mL of non-DRE urine using a patent-pending RNA extraction and reverse transcription methodology.

### 2.2. Pre-Amplification and Quantitative PCR (qPCR)

Using cDNA synthesized from RNA extracted from either DRE or non-DRE urine, MPS2 biomarkers were pre-amplified for 14 cycles (ThermoFisher Scientific, Cat. No. 4391128). Pre-amplified DNA was diluted and used as a template for qPCR. qPCR was performed on the OpenArray^TM^ System using a QuantStudio^TM^ 12K Flex Accufill System and custom OpenArray^TM^ plates containing primers and probes for the 18 MPS2 biomarkers, resulting in relative cycle threshold (Crt) measurements for each analyte.

### 2.3. Determination of Linear Range and Upper Limit of Quantification

Pools of the 18 artificially synthesized MPS2 biomarkers were tested at six concentrations (10^7^, 10^6^, 10^5^, 10^4^, 10^3^, and 10^2^ copies/reaction) to determine the linear range and the upper limit of quantitation (ULOQ) of each MPS2 analyte. Eight replicates of each concentration were run from pre-amplification through qPCR for a total of 48 results. The upper limit of quantification was defined as the highest concentration, with a standard deviation under 0.5 Crt across replicates. The linear range was defined as the range of concentrations within the upper and lower limit of quantification with a qPCR efficiency between 95% and 105% and R^2^ > 0.975.

### 2.4. Determination of Limit of Detection and Lower Limit of Quantification

Pools of the 18 artificially synthesized MPS2 biomarkers were tested at eight concentrations (320, 160, 80, 40, 20, 10, 2.5, 1.25 copies/reaction) to determine the limit of detection (LOD) and the lower limit of quantitation (LLOQ) for each analyte. For both LOD and LLOQ, at least 16 replicates were run of each concentration across 8 runs. The LOD for each analyte was defined as the lowest concentration that could be detected in ≥95% of the replicates. The LLOQ for each analyte was defined as the lowest concentration with a Crt standard deviation ≤0.75 Crt, which is under the previously defined acceptance criteria of a standard deviation under 1 Crt across replicates [10,11].

### 2.5. Precision

The MPS2 precision was measured across several variables including equipment (QuantStudio™ 12K Flex Accufill Systems, VeritiPro™ (ThermoFisher Scientific, Waltham, MA, USA)), operator (Tech A and Tech B), intra-run (repeatability), and inter-run (reproducibility). All precision experiments were performed using a pool of all 18 artificially synthesized MPS2 analytes. Three concentrations of that pool (3200, 1600, 800 copies/reaction) were tested on two VeritiPro™ thermocyclers and two QuantStudio™ 12K Flex Accufill Systems by two technicians over three days across eight replicates. The acceptance criteria included a standard deviation under 0.5 Crt across replicates for each MPS2 analyte.

### 2.6. Interfering Substances

To determine whether certain substances interfere with the MPS2 test, twelve substances that could potentially be found in patient urine were added to contrived samples and tested by MPS2. Contrived samples were created by adding synthesized MPS2 RNA biomarkers to artificial urine (Flinn Scientific, Batavia, IL, USA Catalog Number FB1444) to mimic a clinical urine sample. Note that this artificial urine is synthetically manufactured to mimic human urine and does not include human-derived substances. This approach allowed complete control over the concentration of substances normally found in human urine. For each substance, six replicates were tested. The established acceptance criteria used to rule out interference included an absolute change in the normalized Crt ≤ 0.5 across replicates. The analyte Crts were normalized to the MPS2 reference gene KLK3, which was subsequently used as an input into the MPS2 algorithm [8,9]. Interfering substances experiments were performed using the non-DRE and post-DRE urine methods independently.

### 2.7. MPS2 Score Reproducibility

To determine the non-DRE MPS2 score reproducibility, 30 clinical urine samples (stored at 4 °C) were used to create 10 pools (3 clinical urine samples per pool). Eight replicates of each of the 10 pools were extracted using the non-DRE urine method.

To determine the post-DRE MPS2 score reproducibility, 20 clinical urine samples (stored at 4 °C) were used to create 10 pools (2 clinical urine samples per pool). Eight replicates of each of the 10 pools were extracted using the post-DRE urine method, followed by pre-amplification and quantitative PCR. Fewer clinical urine samples were used to create pools for the post-DRE method compared to the non-DRE method because it requires 1/10th of the urine volume.

MPS2 scores were generated using the qPCR results from the 18 MPS2 biomarkers using a proprietary algorithm [8,9]. The MPS2 score varies from 0 to 100%, representing an individual’s risk for clinically significant prostate cancer, which has been clinically validated for post-DRE and non-DRE urine [8,9].

Since a greater level of reproducibility is necessary at lower MPS2 scores approaching the risk category threshold, the acceptance criteria for reproducibility were designed to scale with the average MPS2 score for each pool. For pools with an MPS2 score ≤25%, the acceptance criteria included a standard deviation ≤5%. For pools with an MPS2 score ≥25%, the acceptance criteria included a standard deviation ≤10%.

## 3. Results

### 3.1. Linear Range and Upper Limit of Quantitation

**Figure 1:**
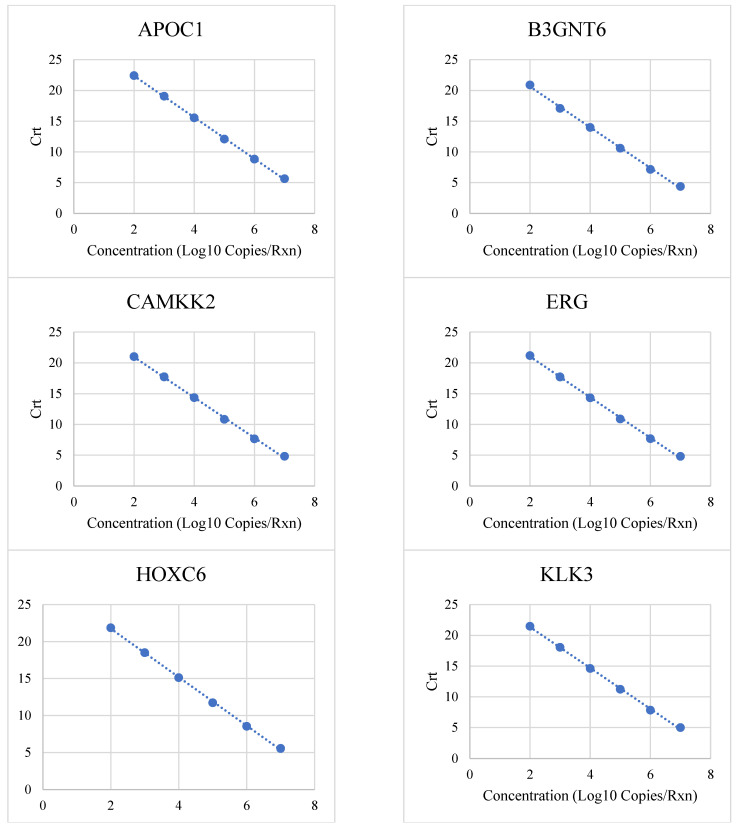

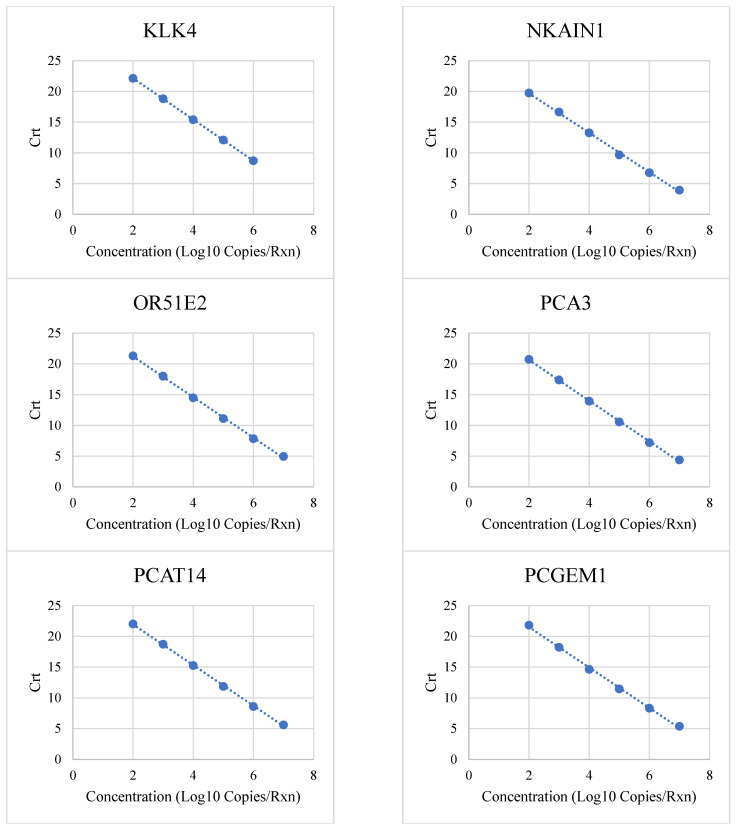

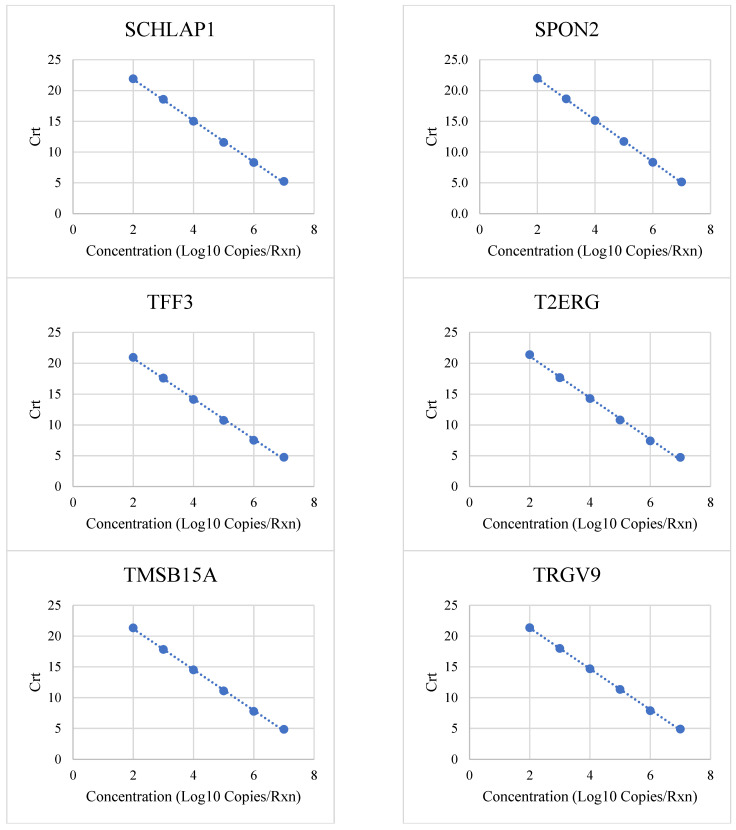

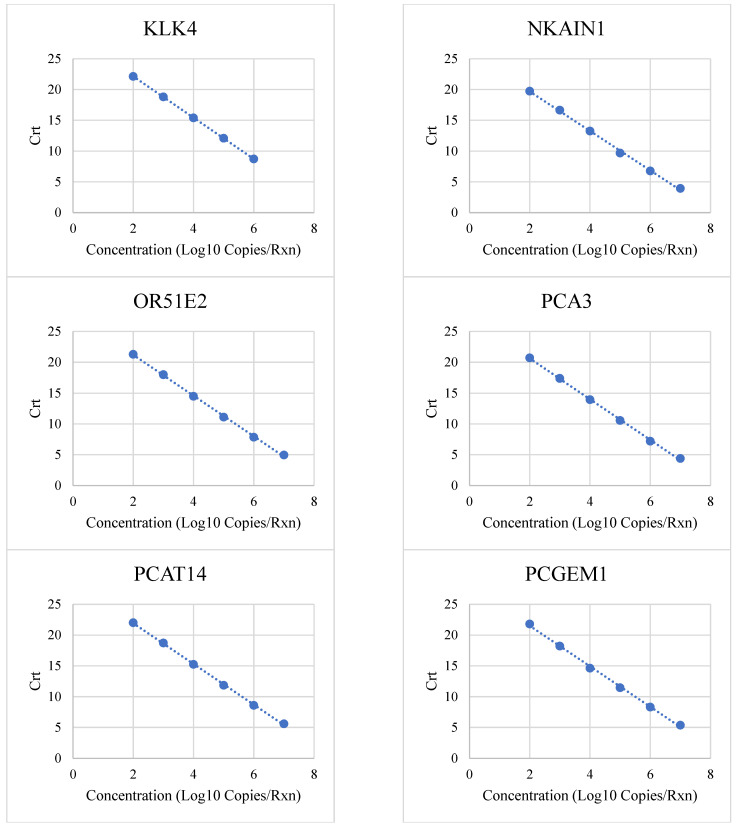

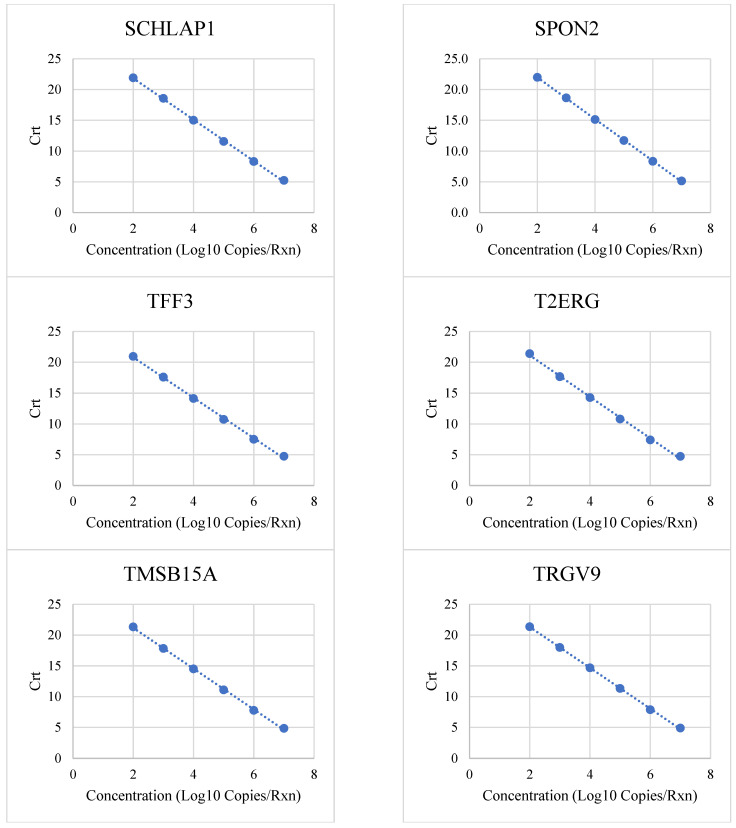
Linear amplification of 18 MPS2 analytes. Dotted line represents the best-fit line by linear regression.

The linear range and upper limit of quantitation were determined by testing six concentrations (10^2^, 10^3^, 10^4^, 10^5^, 10^6^ and 10^7^ copies/reaction). The linear range was defined as the range of concentrations within the upper and lower limit of quantification, with a qPCR efficiency between 95% and 105% and R2 > 0.975. All MPS2 analytes exhibited linear amplification between their individually determined LLOQ and ULOQ (Figure 1, Table 1).

The upper limit of quantitation (ULOQ) was defined as the highest concentration with a standard deviation under 0.5 Crt. All MPS2 analytes except one (17/18) were found to have a ULOQ of 10^7^ copies/reaction with the exception of KLK4, which was determined to be 10^6^ copies per reaction (Table 2). The lower ULOQ for KLK4 was due to template oversaturation, causing no amplification to occur.

### 3.2. Limit of Detection and Lower Limit of Quantitation

The LOD and LLOQ were determined by testing eight concentrations of each MPS2 analyte ranging from 320 down to 1.25 copies/reaction. The LOD for each analyte was defined as the lowest MPS2 analyte concentration that could be detected in ≥95% of the replicates. The LOD for the 18 MPS2 analytes ranges from 40 to 160 copies per reaction (Table 2). The LOD of CAMKK2, KLK4, PCGEM1, SPON2, TFF3, TMSB15A and TRGV9 was determined to be 40 copies/reaction. The LOD of APOC1, B3GNT6, ERG, HOXC6, NKAIN1, OR51E2, PCA3, PCAT144 and T2ERG was determined to be 80 copies/reaction while the LOD of KLK3 and SCHLAP1 was determined to be 160 copies/reaction (Table 2).

The LLOQ for each analyte was defined as the lowest concentration with a standard deviation less than or equal to 0.75 Crt. The LLOQ of ERG, NKAIN1, and SPON2 was determined to be 80 copies/reaction. The LLOQ of APOC1, B3GNT6, CAMKK2, KLK3, PCAT14, PCGEM1, SCHLAP1, TFF3, T2ERG, TMSB15A, and TRGV9 was determined to be 160 copies/reaction. The LLOQ of HOXC6, KLK4, OR51E2, and PCA3 was determined to be 320 copies/reaction with 16 replicates (Table 2).

### 3.3. Precision

Three concentrations (3200, 1600, 800 copies/reaction) of the 18 MPS2 analytes were run by two technicians over three days using two sets of equipment. The concentration range was selected as ten times the LLOQ range for the MPS2 analytes. From this dataset, the overall precision, reproducibility, inter-instrument, and inter-technician precision were analyzed. The acceptance criteria for all precision experiments included a standard deviation under 0.5 Crt, which passed for each MPS2 analyte (Table 3).

The intra-run (repeatability) precision was determined by one experiment run by one technician using one set of equipment over 24 valid results. The intra-run precision passed with a standard deviation ranging from 0.1 to 0.3 Crt (Table 3). The inter-run (reproducibility) precision was determined by three experiments run by one technician over three days using one set of equipment over 71 valid results. The inter-run precision passed with a standard deviation ranging from 0.1 to 0.3 Crt (Table 3). The inter-instrument (QuantStudio^TM^ 12K Flex Accufill System) precision was determined by two experiments run by one technician over two days using two different QuantStudio^TM^ 12K Flex Accufill Systems across 48 valid results. The inter-instrument (QuantStudio^TM^ 12K Flex Accufill System) precision passed with a standard deviation ranging from 0.1 to 0.3 Crt (Table 3). The inter-instrument (VeritiPro^TM^) precision was determined by three experiments run using two different VeritiPro^TM^ thermocyclers across 95 valid results. The inter-instrument (VeritiPro^TM^) precision passed with a standard deviation ranging from 0.1 to 0.3 Crt (Table 3). The inter-technician precision was determined by two experiments run by two technicians over two days using the same equipment across 48 valid results. The inter-technician precision passed with a standard deviation ranging from 0.1 to 0.3 Crt (Table 3).

### 3.4. Interfering Substances

With both the non-DRE and post-DRE urine extraction methods, 11 out of the 12 potentially interfering substances tested did not interfere with the MPS2 analyte detection, resulting in an absolute change in the KLK3 normalized Crt ≤ 0.5 compared to the controls (varied between −0.5 and 0.4). Negative KLK3 normalized Crt occurs when the Crt of an analyte of interest decreases more than KLK3 when comparing the control to the result of the interfering substances. Positive KLK3 normalized Crt occurs when the Crt of the analyte of interest decreases less than KLK3 when comparing the control to the result for the interfering substances. Whole blood did interfere with MPS2 analyte detection in both the non-DRE and post-DRE urine extraction methods. With the non-DRE method, 0.05% whole blood caused significant interference in MPS2 analyte detection with a change in the KLK3-normalized Crt between −2.7 and 5.1 Crt (Table 4). With the post-DRE method, 0.05% whole blood again caused significant interference in MPS2 analyte detection, with a change in normalized Crt between −1.0 and 8.5 Crt (Table 5).

### 3.5. MPS2 Score Reproducibility (Non-DRE and Post-DRE Methods)

Pooled urine samples, generated using remnant clinical samples, were tested across eight replicates to investigate the reproducibility of the MPS2 score. Importantly, these urine pools spanned a large range of MPS2 scores from 3% to 82% (Table 6). The MPS2 scores generated using the non-DRE and post-DRE urine methods were highly reproducible, with an MPS2 score standard deviation across 20 samples ranging from 1% to 7% over eight replicates (Table 6). The average MPS2 score standard deviation was 3% and 4% for the non-DRE and post-DRE urine extraction methods, respectively. The acceptance criteria were designed to require a greater level of reproducibility at lower MPS2 scores approaching the risk category threshold. All pools passed the acceptance criteria, with all pools with an average MPS2 score ≤ 25% having a standard deviation ≤5%, and all pools with an average MPS2 score ≥ 25% having a standard deviation ≤10%.

## 4. Discussion

In this study, we have comprehensively evaluated the ability of MPS2 to reproducibly measure target MPS2 analytes. The limits of reproducible MPS2 analyte detection were analytically determined based on Clinical & Laboratory Standards Institute (CLSI) guidelines and analytical validations performed on similarly designed prostate cancer diagnostic screening tests [10,12,13,14,15]. The limit of detection for MPS2 analytes is in a similar range as other urine-based prostate cancer screening tests, with the lower limit of detection of MPS2 analytes ranging from 40 to 160 copies per reaction and an upper limit of quantitation ranging between 10^6^ and 10^7^ copies per reaction [16,17]. MPS2 analyte detection in this range is highly linear (R^2^ ≥ 0.98) with PCR efficiencies ranging from 97% to 105%, indicating negligible amplification bias.

The robustness and reproducibility of MPS2 analyte detection was measured within runs as well as across time, technicians, pre-amplification thermal cyclers (VeritiPro^TM^) and real-time PCR systems (QuantStudio12K Flex OpenArray System). MPS2 analyte signals were highly reproducible across these factors at three concentrations, illustrating the robustness of MPS2 for clinical testing.

Finally, the impact of twelve substances that could occur in clinical urine samples was tested on MPS2 analyte detection. All substances were tested at elevated concentrations compared to what would typically be observed in human urine. Eleven of the twelve substances tested had negligible MPS2 analyte detection. The only substance that had a substantial impact was whole blood, tested at a 0.05% concentration, which significantly reduced MPS2 analyte signals. Whole blood and plasma have been shown to be rich in RNases, with RNase activity in the range of 0.1 to 1.0 μg/mL [18]. It is likely that the reduced MPS2 analyte signals observed when whole blood is added to urine samples is due to these RNase degrading the urinary RNA, but the direct inhibition of whole blood components on MPS2 extraction and potential carry-over into downstream reactions remain a possibility. The reduction in the MPS2 analyte signal due to hematuria would likely result in an invalid sample result, due to insufficient analyte detection. Hematuria rates in men have been investigated in several studies. In one study evaluating hematuria rates for men visiting their general practitioner from 1993 to 1994, 4 of 1000 patients were positive for hematuria [19]. More recently, a retrospective analysis of incidental hematuria findings in routine urinalysis from 2008 to 2013 discovered that 8.6% of patients had positive hematuria [20]. To the best of our knowledge, the hematuria rate in men with prostate cancer has not been directly studied; however, it has been shown that the prevalence of prostate cancer in men with hematuria is 3% [21]. Given the impact of blood on MPS2 performance and the estimated prevalence of hematuria in the population, clinical samples are tested for blood as part of regular MPS2 clinical testing.

This analytical validation confirms the robustness of MPS2 to accurately and reproducibly detect the 18 MPS2 target analytes. In conjunction with the previous clinical validations [8,9], this work further shows that the biomarker signals measured in MPS2 are robust and can be used clinically to predict clinically significant prostate cancer.

## Figures and Tables

**Table 1:** MPS2 linear amplification metrics.

Target	Low (Copies/Rxn)	High (Copies/Rxn)	R^2^	PCR Efficiency
APOC1	100	10,000,000	0.9997	98%
B3GNT6	100	10,000,000	0.9986	101%
CAMKK2	100	10,000,000	0.999	102%
ERG	100	10,000,000	0.999	101%
HOXC6	100	10,000,000	0.9994	102%
KLK3	100	10,000,000	0.9991	100%
KLK4	100	1,000,000	1	99%
NKAIN1	100	10,000,000	0.9985	105%
OR51E2	100	10,000,000	0.9992	101%
PCA3	100	10,000,000	0.9991	101%
PCAT14	100	10,000,000	0.9995	101%
PCGEM1	100	10,000,000	0.98	98%
SCHLAP1	100	10,000,000	0.9994	98%
SPON2	100	10,000,000	0.9998	97%
TFF3	100	10,000,000	0.9988	102%
T2ERG	100	10,000,000	0.998	98%
TMSB15A	100	10,000,000	0.9994	100%
TRGV9	100	10,000,000	0.9997	100%

**Table 2:** MPS2 analyte LOD, LLOQ and ULOQ.

MPS2 Analyte	LOD in Copies/Reaction (% Detection)	LLOQ in Copies/Reaction (SD)	ULOQ in Copies/Reaction (SD)
APOC1	80 (100%)	160 (0.43)	10,000,000 (0.17)
B3GNT6	80 (100%)	160 (0.53)	10,000,000 (0.08)
CAMKK2	40 (95%)	160 (0.45)	10,000,000 (0.08)
ERG	80 (97%)	80 (0.69)	10,000,000 (0.06)
HOXC6	80 (100%)	320 (0.43)	10,000,000 (0.07)
KLK3	160 (100%)	160 (0.53)	10,000,000 (0.06)
KLK4	40 (100%)	320 (0.40)	1,000,000 (0.34)
NKAIN1	80 (97%)	80 (0.59)	10,000,000 (0.16)
OR51E2	80 (100%)	320 (0.27)	10,000,000 (0.13)
PCA3	80 (100%)	320 (0.33)	10,000,000 (0.10)
PCAT14	80 (98%)	160 (0.45)	10,000,000 (0.12)
PCGEM1	40 (95%)	160 (0.46)	10,000,000 (0.11)
SCHLAP1	160 (100%)	160 (0.74)	10,000,000 (0.12)
SPON2	40 (95%)	80 (0.60)	10,000,000 (0.06)
TFF3	40 (95%)	160 (0.37)	10,000,000 (0.05)
T2ERG	80 (100%)	160 (0.69)	10,000,000 (0.04)
TMSB15A	40 (95%)	160 (0.53)	10,000,000 (0.08)
TRGV9	40 (96%)	160 (0.35)	10,000,000 (0.12)

**Table 3:** MPS2 analyte precision. Data represent the Crt mean and standard deviation for each MPS2 analyte.

Target	Copies/Reaction	Overall Precision	Intra-Run (Repeatability)	Inter-Run (Reproducibility)	Inter-Instrument (QuantStudio)	Inter-Instrument (VeritiPro)	Inter-Technician
APOC1	3200	16.6 ± 0.2	16.6 ± 0.2	16.7 ± 0.2	16.5 ± 0.2	16.7 ± 0.1	16.7 ± 0.2
	1600	17.8 ± 0.2	17.9 ± 0.1	17.9 ± 0.2	17.9 ± 0.1	17.8 ± 0.2	17.8 ± 0.2
	800	18.8 ± 0.3	18.8 ± 0.2	18.9 ± 0.2	18.8 ± 0.2	18.8 ± 0.3	18.9 ± 0.2
B3GNT6	3200	15.5 ± 0.2	15.3 ± 0.1	15.6 ± 0.2	15.3 ± 0.1	15.6 ± 0.2	15.5 ± 0.3
	1600	16.7 ± 0.2	16.7 ± 0.1	16.8 ± 0.1	16.7 ± 0.1	16.7 ± 0.2	16.8 ± 0.1
	800	17.6 ± 0.3	17.6 ± 0.2	17.8 ± 0.2	17.5 ± 0.2	17.6 ± 0.3	17.7 ± 0.3
CAMKK2	3200	15.7 ± 0.2	15.5 ± 0.1	15.7 ± 0.2	15.5 ± 0.1	15.7 ± 0.2	15.7 ± 0.2
	1600	16.8 ± 0.2	16.8 ± 0.1	16.9 ± 0.1	16.8 ± 0.1	16.9 ± 0.2	16.9 ± 0.1
	800	17.8 ± 0.2	17.7 ± 0.2	17.9 ± 0.2	17.7 ± 0.1	17.8 ± 0.2	17.9 ± 0.2
ERG	3200	15.7 ± 0.2	15.6 ± 0.1	15.8 ± 0.2	15.6 ± 0.1	15.8 ± 0.2	15.7 ± 0.2
	1600	16.9 ± 0.2	16.9 ± 0.1	17.0 ± 0.1	16.9 ± 0.1	17.0 ± 0.2	17.0 ± 0.1
	800	17.8 ± 0.3	17.8 ± 0.2	18.0 ± 0.2	17.8 ± 0.1	17.9 ± 0.3	18 ± 0.2
HOXC6	3200	16.3 ± 0.2	16.2 ± 0.2	16.4 ± 0.2	16.1 ± 0.2	16.4 ± 0.2	16.3 ± 0.2
	1600	17.6 ± 0.2	17.6 ± 0.1	17.6 ± 0.1	17.6 ± 0.1	17.6 ± 0.2	17.6 ± 0.1
	800	18.4 ± 0.3	18.5 ± 0.2	18.6 ± 0.2	18.4 ± 0.2	18.5 ± 0.3	18.6 ± 0.2
KLK3	3200	16.0 ± 0.2	15.8 ± 0.1	16.1 ± 0.2	15.8 ± 0.1	16.1 ± 0.2	16 ± 0.2
	1600	17.3 ± 0.2	17.3 ± 0.2	17.4 ± 0.1	17.3 ± 0.2	17.3 ± 0.2	17.4 ± 0.2
	800	18.1 ± 0.3	18.2 ± 0.2	18.3 ± 0.2	18.1 ± 0.2	18.2 ± 0.3	18.3 ± 0.2
KLK4	3200	16.7 ± 0.2	16.6 ± 0.2	16.7 ± 0.1	16.5 ± 0.2	16.7 ± 0.1	16.7 ± 0.2
	1600	18.0 ± 0.2	18.0 ± 0.1	18.0 ± 0.1	18.0 ± 0.1	18.0 ± 0.2	18.0 ± 0.1
	800	18.9 ± 0.3	18.9 ± 0.3	19.1 ± 0.2	18.9 ± 0.3	19.0 ± 0.3	19.0 ± 0.3
NKAIN1	3200	14.5 ± 0.2	14.4 ± 0.3	14.4 ± 0.2	14.3 ± 0.2	14.5 ± 0.2	14.4 ± 0.2
	1600	15.7 ± 0.2	15.8 ± 0.1	15.7 ± 0.1	15.8 ± 0.1	15.7 ± 0.2	15.7 ± 0.1
	800	16.5 ± 0.3	16.6 ± 0.2	16.7 ± 0.2	16.6 ± 0.2	16.5 ± 0.3	16.6 ± 0.2
OR51E2	3200	15.7 ± 0.1	15.6 ± 0.1	15.7 ± 0.1	15.6 ± 0.1	15.7 ± 0.1	15.7 ± 0.1
	1600	16.9 ± 0.1	17.0 ± 0.1	17.0 ± 0.1	17.0 ± 0.1	17.0 ± 0.1	17.0 ± 0.1
	800	17.8 ± 0.2	17.9 ± 0.2	18.0 ± 0.2	17.8 ± 0.2	17.9 ± 0.2	17.9 ± 0.2
PCA3	3200	15.5 ± 0.2	15.3 ± 0.2	15.6 ± 0.2	15.3 ± 0.2	15.5 ± 0.2	15.5 ± 0.2
	1600	16.7 ± 0.2	16.7 ± 0.1	16.8 ± 0.1	16.7 ± 0.1	16.7 ± 0.2	16.8 ± 0.1
	800	17.7 ± 0.3	17.7 ± 0.2	17.9 ± 0.2	17.7 ± 0.2	17.7 ± 0.3	17.8 ± 0.2
PCAT14	3200	16.6 ± 0.2	16.4 ± 0.1	16.6 ± 0.2	16.5 ± 0.1	16.6 ± 0.2	16.6 ± 0.2
	1600	17.8 ± 0.2	17.8 ± 0.1	17.8 ± 0.1	17.8 ± 0.1	17.8 ± 0.2	17.8 ± 0.1
	800	18.7 ± 0.2	18.6 ± 0.2	18.8 ± 0.2	18.6 ± 0.2	18.7 ± 0.2	18.8 ± 0.2
PCGEM1	3200	16.4 ± 0.2	16.2 ± 0.1	16.5 ± 0.2	16.2 ± 0.1	16.4 ± 0.2	16.4 ± 0.2
	1600	17.6 ± 0.2	17.7 ± 0.1	17.7 ± 0.1	17.7 ± 0.1	17.6 ± 0.2	17.7 ± 0.1
	800	18.5 ± 0.3	18.5 ± 0.2	18.7 ± 0.2	18.5 ± 0.2	18.6 ± 0.3	18.7 ± 0.3
SCHLAP1	3200	16.3 ± 0.2	16.2 ± 0.1	16.4 ± 0.2	16.2 ± 0.1	16.4 ± 0.2	16.3 ± 0.2
	1600	17.6 ± 0.2	17.6 ± 0.1	17.7 ± 0.1	17.6 ± 0.1	17.6 ± 0.2	17.6 ± 0.1
	800	18.4 ± 0.3	18.4 ± 0.2	18.6 ± 0.2	18.4 ± 0.2	18.5 ± 0.3	18.5 ± 0.2
SPON2	3200	16.6 ± 0.2	16.4 ± 0.1	16.6 ± 0.2	16.4 ± 0.2	16.6 ± 0.2	16.6 ± 0.2
	1600	17.8 ± 0.2	17.9 ± 0.1	17.9 ± 0.2	17.8 ± 0.1	17.9 ± 0.2	17.9 ± 0.2
	800	18.8 ± 0.3	18.8 ± 0.2	19.0 ± 0.2	18.8 ± 0.2	18.9 ± 0.3	19.0 ± 0.3
TFF3	3200	15.6 ± 0.1	15.5 ± 0.1	15.6 ± 0.2	15.5 ± 0.1	15.6 ± 0.2	15.6 ± 0.2
	1600	16.8 ± 0.2	16.8 ± 0.1	16.8 ± 0.1	16.8 ± 0.1	16.8 ± 0.2	16.9 ± 0.1
	800	17.7 ± 0.2	17.7 ± 0.2	17.8 ± 0.2	17.6 ± 0.2	17.7 ± 0.2	17.8 ± 0.2
T2ERG	3200	15.4 ± 0.2	15.1 ± 0.2	15.4 ± 0.3	15.2 ± 0.2	15.4 ± 0.2	15.4 ± 0.3
	1600	16.6 ± 0.2	16.6 ± 0.2	16.7 ± 0.1	16.6 ± 0.1	16.6 ± 0.2	16.7 ± 0.2
	800	17.5 ± 0.3	17.4 ± 0.2	17.7 ± 0.2	17.4 ± 0.2	17.6 ± 0.3	17.6 ± 0.3
TMSB15A	3200	15.8 ± 0.2	15.7 ± 0.1	15.9 ± 0.2	15.7 ± 0.1	15.9 ± 0.2	15.8 ± 0.2
	1600	17.1 ± 0.2	17.1 ± 0.1	17.2 ± 0.1	17.1 ± 0.1	17.1 ± 0.2	17.2 ± 0.2
	800	17.9 ± 0.3	17.9 ± 0.2	18.1 ± 0.2	17.9 ± 0.2	18.0 ± 0.3	18.0 ± 0.3
TRGV9	3200	16.0 ± 0.2	15.9 ± 0.1	16.1 ± 0.2	15.9 ± 0.1	16.1 ± 0.2	16.0 ± 0.2
	1600	17.3 ± 0.2	17.3 ± 0.2	17.4 ± 0.2	17.3 ± 0.2	17.3 ± 0.2	17.4 ± 0.2
	800	18.2 ± 0.3	18.1 ± 0.3	18.4 ± 0.3	18.1 ± 0.2	18.2 ± 0.3	18.3 ± 0.3

**Table 4:** MPS2 interfering substance assessment using the non-DRE urine extraction method. Data represent the difference in normalized Crt between the control urine and the test substance.

Substance	Concentration	APOC1	B3GNT6	CAMKK2	ERG	HOXC6	KLK3	KLK4	NKAIN1	OR51E2	PCA3	PCAT14	PCGEM1	SCHLAP1	SPON2	TFF3	**T2ERG**	**TMSB15A**	**TRGV9**
Whole Blood	0.05%	0.0	3.1	−0.3	−2.7	1.5	0.0	0.4	−0.6	1.4	3.6	0.7	−1.1	−4.4	5.1	1.8	0.2	3.1	0.8
Alcohol (100% ethanol)	2.5%	0.2	0.0	0.1	−0.2	0.0	0.0	0.1	0.2	0.2	0.0	0.0	0.1	−0.3	0.0	−0.1	−0.2	−0.3	−0.2
Triglycerides	15,000 mg/L	0.4	0.0	0.1	0.2	0.1	0.0	0.2	0.3	0.2	0.1	0.0	0.2	0.1	0.0	−0.1	−0.1	−0.1	0.1
Hemoglobin	2000 mg/L	0.0	0.3	0.0	0.0	−0.1	0.0	0.2	−0.1	0.1	0.1	0.0	0.0	0.1	−0.1	−0.2	0.1	−0.1	0.0
Calcium (CaCl)	800 mg/L	0.0	0.3	0.3	0.1	−0.1	0.0	0.2	0.2	0.0	0.1	0.0	0.0	0.1	−0.1	−0.1	0.1	−0.1	0.1
Cholesterol	700 mg/L	0.2	0.3	0.2	0.1	−0.1	0.0	0.2	0.2	0.0	0.1	0.0	0.2	0.0	−0.1	−0.1	0.0	−0.2	−0.1
Glucose	400 mg/L	−0.2	0.1	0.0	0.0	−0.2	0.0	0.0	−0.1	0.0	0.0	−0.2	0.0	−0.3	−0.3	−0.3	−0.2	−0.3	−0.1
Sodium (NaCl)	8000 mg/L	−0.1	0.2	0.1	0.1	−0.1	0.0	0.1	0.0	0.0	0.1	−0.1	−0.1	−0.1	−0.1	−0.2	0.1	−0.2	0.0
Albumin	400 mg/L	−0.5	−0.2	0.0	−0.5	−0.3	0.0	−0.4	−0.2	0.0	0.2	0.0	−0.1	−0.1	−0.4	0.2	−0.4	−0.2	−0.4
Microorganisms	257 cfu/mL	0.0	0.2	0.1	0.0	−0.2	0.0	−0.1	0.0	0.0	0.1	−0.1	0.1	0.2	−0.1	0.0	0.0	−0.1	−0.1
Free Bilirubin	60 mg/L	0.2	0.0	0.1	0.1	0.1	0.0	0.2	0.0	0.1	−0.1	0.0	−0.2	0.1	0.1	−0.2	0.3	0.2	0.1
Total Proteins	1 mg/L	0.1	0.1	0.1	0.0	0.1	0.0	0.1	0.1	−0.1	−0.1	0.0	0.0	0.2	0.1	0.0	0.2	0.2	0.0

**Table 5:** MPS2 interfering substance assessment using the post-DRE urine extraction method. Data represent the difference in normalized Crt between the control urine and the test substance.

Substance	Concentration	APOC1	B3GNT6	CAMKK2	ERG	HOXC6	KLK3	KLK4	NKAIN1	OR51E2	PCA3	PCAT14	PCGEM1	SCHLAP1	SPON2	TFF3	T2ERG	TMSB15A	TRGV9
Whole Blood	0.05%	1.0	0.4	1.8	−1.0	1.0	0.0	0.5	0.2	1.4	2.7	0.7	0.3	−0.6	8.5	3.1	1.1	0.9	4.2
Alcohol (100% ethanol)	2.5%	−0.1	−0.5	−0.1	−0.1	0.1	0.0	0.0	−0.3	−0.3	−0.2	−0.2	−0.2	−0.1	−0.2	−0.3	0.1	0.0	0.0
Triglycerides	15,000 mg/L	−0.1	−0.3	−0.1	−0.1	−0.1	0.0	−0.4	0.0	−0.1	0.0	0.1	0.0	−0.1	0.0	−0.1	0.0	0.1	0.1
Hemoglobin	2000 mg/L	−0.1	−0.1	−0.1	−0.1	0.0	0.0	−0.1	0.1	0.0	0.0	0.2	−0.1	0.0	−0.1	−0.2	0.3	0.3	0.1
Calcium (CaCl)	800 mg/L	−0.1	−0.3	−0.2	0.0	−0.1	0.0	−0.2	0.2	0.0	−0.1	0.1	0.0	0.2	−0.1	−0.1	0.1	0.3	0.1
Cholesterol	700 mg/L	−0.4	−0.4	−0.2	−0.1	0.0	0.0	−0.3	−0.1	−0.4	0.0	0.0	−0.2	−0.2	−0.1	−0.1	0.1	0.0	0.0
Glucose	400 mg/L	−0.2	−0.3	−0.1	−0.1	0.0	0.0	−0.1	−0.1	−0.2	0.0	0.0	−0.1	−0.3	0.2	0.0	0.1	−0.1	0.1
Sodium (NaCl)	8000 mg/L	−0.2	−0.2	0.0	0.2	0.2	0.0	0.0	0.0	0.0	0.2	0.3	0.0	0.2	0.1	0.0	−0.3	0.2	0.3
Albumin	400 mg/L	−0.3	−0.1	0.2	−0.1	−0.1	0.0	−0.1	0.1	0.3	0.3	0.3	0.1	−0.3	0.1	0.3	0.4	0.2	0.2
Microorganisms	257 cfu/mL	−0.1	−0.1	0.0	0.1	0.0	0.0	−0.1	0.1	0.1	0.0	0.1	0.0	−0.1	0.0	−0.1	−0.2	0.0	0.3
Free Bilirubin	60 mg/L	−0.1	−0.3	−0.1	−0.1	0.1	0.0	−0.2	−0.2	−0.1	−0.1	0.0	−0.1	−0.1	0.0	−0.1	0.2	0.2	0.4
Total Proteins	1 mg/L	−0.3	−0.2	−0.2	−0.1	−0.1	0.0	−0.1	−0.1	−0.3	−0.1	0.0	0.0	−0.1	0.0	−0.1	0.1	0.1	−0.1

**Table 6:** The reproducibility of the MPS2 score of 10 urine pools across 8 replicates using the non-DRE and post-DRE Urine Extraction Method.

RNA Extraction Method	Pool	MPS2 Score (Mean ± SD)
non-DRE Urine	1	40% ± 4%
	2	82% ± 2%
	3	9% ± 1%
	4	7% ± 3%
	5	38% ± 5%
	6	79% ± 4%
	7	3% ± 2%
	8	63% ± 4%
	9	4% ± 2%
	10	28% ± 4%
post-DRE Urine	1	5% ± 1%
	2	48% ± 7%
	3	61% ± 4%
	4	73% ± 3%
	5	23% ± 5%
	6	11% ± 3%
	7	19% ± 4%
	8	7% ± 2%
	9	26% ± 6%
	10	7% ± 2%

## Data Availability

The raw data supporting the conclusions of this article will be made available by the authors on request.

## Abbreviations

The following abbreviations are used in this manuscript:

Crt	Relative cycle threshold
csPCa	Clinically significant prostate cancer
DRE	Digital rectal exam
LLOQ	Lower limit of quantitation
LOD	Limit of detection
MPS2	MyProstateScore 2.0
PCa	Prostate cancer
PSA	Prostate-specific antigen
qPCR	Quantitative polymerase chain reaction
ULOQ	Upper limit of quantitation
USPSTF	United States Preventive Services Task Force
